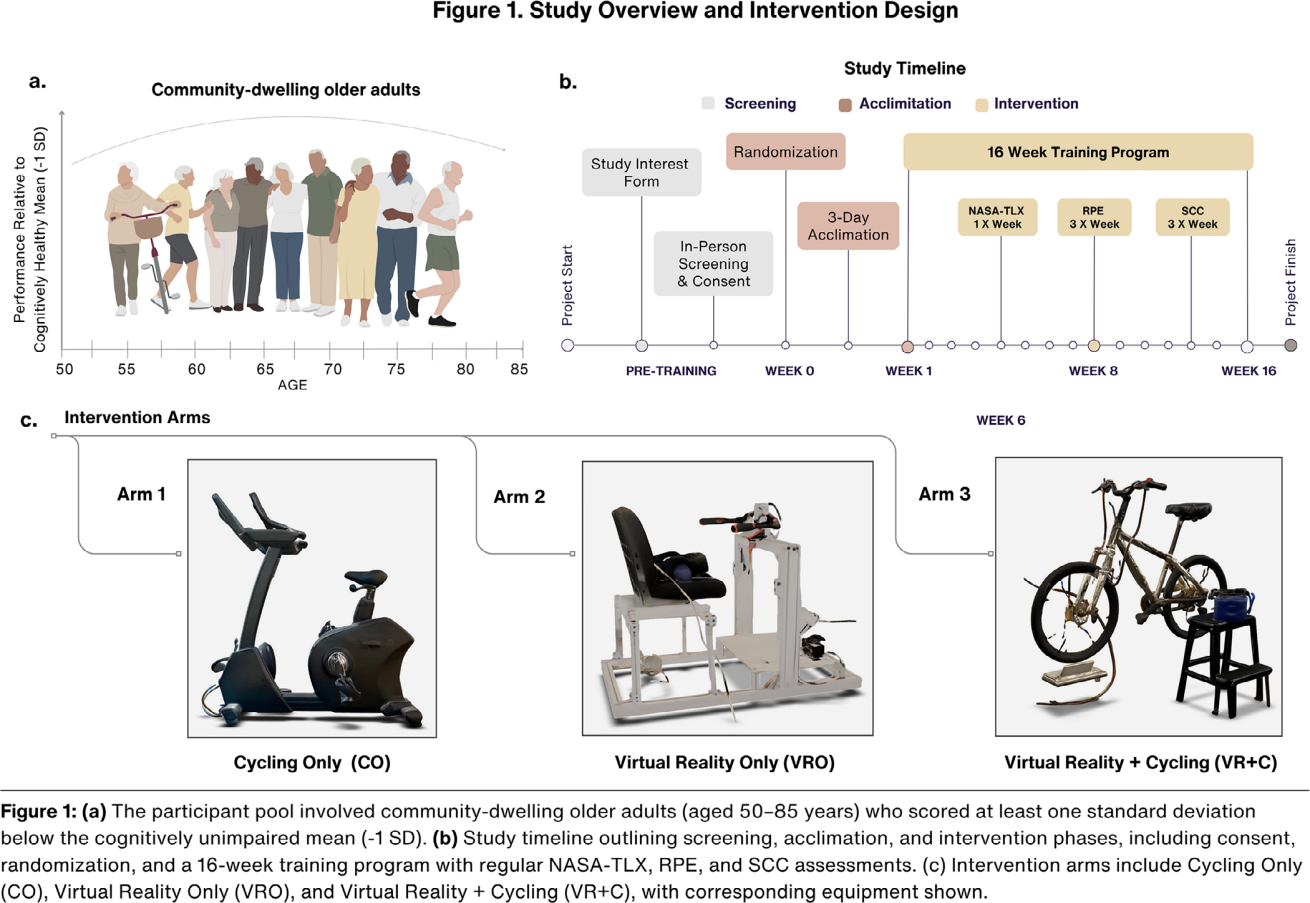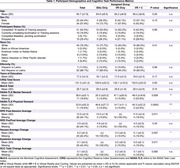# Combined Virtual Reality and Physical Activity Mitigates Mental Workload, Physical Exertion, and Simulator Sickness Compared to Virtual Reality Only in Older Adults

**DOI:** 10.1002/alz70863_110518

**Published:** 2025-12-23

**Authors:** Cynthia Angela Nyongesa, Kimberly Espejo, Marcella Barneclo, Emily Ager, Roshan Ravichandran, Adam Gardner, Joshua A Fierro, Yilei Dong, Joanna L Eckhardt, Edwin D Sookiassian, A. Lisette Isenberg, Ashwin Sakhare, Judy Pa

**Affiliations:** ^1^ Alzheimer's Disease Cooperative Study (ADCS), University of California, San Diego, La Jolla, CA USA; ^2^ Neurosciences Graduate Program, University of California, San Diego, La Jolla, CA USA

## Abstract

**Background:**

Virtual reality (VR) interventions offer immersive cognitive training experiences for older adults; however, challenges such as simulator sickness and cognitive workload can impact participation and adherence. Integrating physical activity, such as stationary cycling, with VR may alleviate these challenges and improve engagement.

**Methods:**

Sixty‐three community‐dwelling older adults (ages 50–85) were randomized into three groups: VR‐Only (VRO), Cycling‐Only (CO), and VR with Cycling (VR+C). Assessments included the NASA Task Load Index (NASA‐TLX) to measure cognitive workload, the Borg Rating of Perceived Exertion (RPE) to assess subjective physical effort, and the Short Symptom Checklist (SSC) to evaluate simulator sickness symptoms. Data was collected weekly for 16 weeks and analyzed using mixed‐effects models and repeated measures ANOVA to examine changes over time and between groups.

**Results:**

Participants in the VRO group exhibited significantly higher simulator sickness and cognitive workload compared to the VR+C group, with notable increases in nausea (p < 0.001), eyestrain (p < 0.001), and dizziness (p < 0.01). Analysis of symptom severity trends revealed that the VRO group experienced peak discomfort around week 8, indicating a critical adaptation period. In contrast, the VR+C group showed a more gradual increase in discomfort with fewer symptom spikes. The CO group reported the highest perceived exertion but lower cognitive workload compared to the VRO group, as expected. Longitudinal analysis demonstrated a steady rise in perceived effort for the CO group while the VR+C group adapted more gradually over time. Correlation analysis showed a significant relationship between physical demand and perceived exertion (r = 0.64, *p* < 0.001), suggesting adaptation effects. Adherence rates were highest in the VRO group (80.95%), followed by CO (75%), and lowest in VR+C (70%), indicating that physical effort perception plays a role in retention.

**Conclusion:**

Integrating stationary cycling with VR‐based cognitive training presents a promising strategy to enhance cognitive resilience while reducing simulator sickness and cognitive overload in older adults. Findings suggest that multimodal interventions can provide an effective approach to supporting cognitive health, with the potential to enhance long‐term adherence and play a pivotal role in early exercise and VR interventions for individuals at risk of Alzheimer's disease and related dementias.